# Dr. H. De V. Pratt

**Published:** 1899-02

**Authors:** 


Dr. H. De V. Pratt, of Elmira, one of the most prominent physicians in Chemung County, died of pneumonia at his home in that city January 12, 1899, aged 42 years. He was a member of many fraternal societies, a prominent member of the Medical Society of the State of New York, of the Elmira Academy of Medicine and of the Medical Society of the County of Chemung, and was formerly surgeon of the 30th Separate Company. His name was on the program of the Medical Society of the State of New York for its next annual meeting.

